# Ultrasound Urodynamics: A Review of Ultrasound Imaging Techniques for Enhanced Bladder Functional Diagnostics

**DOI:** 10.1007/s11884-024-00758-2

**Published:** 2024-07-10

**Authors:** Brendan McCormack, Hailey L. Hampton, John E. Speich, Stephen C. Radley, Linda S. Burkett, Adam P. Klausner

**Affiliations:** 1 Department of Surgery/Division of Urology, Virginia Commonwealth University School of Medicine, Richmond, VA USA; 2 Department of Mechanical & Nuclear Engineering, Virginia Commonwealth University College of Engineering, Richmond, VA USA; 3 Department of Urogynaecology, Sheffield Teaching Hospitals NHS Foundation Trust, Sheffield, UK; 4 Department of Obstetrics and Gynecology, Virginia Commonwealth University School of Medicine, Richmond, VA USA

**Keywords:** Ultrasound, Overactive Bladder, Urodynamics

## Abstract

**Purpose of Review:**

Invasive urodynamics are currently used to diagnose disorders of bladder function. However, due to patient discomfort as well as artifacts induced by catheters and non-physiologic filling, less invasive screening tools that can improve diagnostic information, such as ultrasound are required. The purpose of this review is to assess different modalities of ultrasound as applied to functional bladder imaging. This information will help guide future studies in the use of ultrasound during urodynamics.

**Recent Findings:**

Recently, multiple studies have employed ultrasound to evaluate bladder volume, wall thickness, shape, vibrometry, elastography, compliance, biomechanics, and micromotion during urodynamics. These new techniques have used both 2D and 3D ultrasound techniques to evaluate bladder changes during filling. Continued research is needed to confirm ongoing findings prior to widespread incorporation into clinical practice.

**Summary:**

This review demonstrates the potential use of ultrasound as an adjunct to urodynamics for the diagnostic evaluation of functional bladder disorders.

## Introduction

### Why Standard Multichannel Urodynamics Are Not Good Enough

Historically, multichannel urodynamics (**UDS**) has been considered the gold standard for the assessment of lower urinary tract function in individuals with various forms of voiding and storage dysfunction including neurogenic lower urinary tract dysfunction, incomplete emptying, urinary retention, urinary incontinence, and medication-refractory overactive bladder (**OAB**). Specifically, the purpose of UDS is to determine underlying or contributory causes such as involuntary detrusor contractions, the presence of bladder outlet obstruction, the identification of a poorly contractile detrusor muscle, poor compliance and the finding of detrusor-sphincter dyssynergia.[1] Although UDS is designed to replicate a patient’s typical filling and voiding cycles, the nature of the testing, usually with supra-physiologic filling and the required placement of invasive urethral and rectal/vaginal catheters, can limit the quality and consistency of the results.[2, 3] In addition, patients report embarrassment and even humiliation during UDS, and the test is expensive and associated with complications including pain, bleeding, and urinary tract infections.[4].

Furthermore, UDS results are poorly repeatable and insensitive, particularly in the detection of detrusor overactivity. Our current nomenclature is also problematic, as at present, detrusor overactivity is exclusively a urodynamic condition. Abnormal contractions detected by other means, including ultrasound, therefore require alternative nomenclature, such as involuntary detrusor activity or a reclassification to indicate the detection modality. Gupta and colleagues found poor reproducibility of UDS both between fills during the same study and in repeat visits, likely due to physiological fluctuation in bladder function and inherent lack of sensitivity in testing instruments.[3] There is also the issue of study quality. The International Continence Society (**ICS**) has published standardization documents regarding appropriate UDS techniques.[1, 19, 20] However, residency training programs, especially in the U.S., provide limited in-depth UDS training (only 10 procedures are required to achieve the required UDS case minimum). The market often drives the type of equipment utilized, and ICS standardization documents are sometimes not reflective of actual practice patterns. For example, most U.S. practices use the air-charged (T-Doc®) urethral catheters. However, the ICS documents do not definitively support this as an acceptable standard.[1, 19].

Finally, development and refinement of current UDS techniques often requires studies involving normal, healthy volunteers as control subjects. Unfortunately, several groups have reported high rates of artifact in these studies and caution against making interpretive comparisons using UDS data from normal, healthy volunteers as control subjects.[21, 22] Although UDS has been considered the gold standard, it simply is not good enough. So, to improve on this relatively low bar, investigators have explored a wide variety of new technologies and refinements.[23] The current review will focus on the use of ultrasound imaging for improved UDS diagnostics. Several areas that will be highlighted include ultrasound as a measure of bladder volume, wall thickness, shape, vibrometry, elastography, compliance, biomechanics, and micromotion (Table 1). Although ultrasound techniques have been employed in other areas of the lower urinary tract including the pelvic floor muscles and urethra, this review will focus specifically on the use of ultrasound in the assessment of bladder function.

**Table 1:** Ultrasound Urodynamic Metrics

Metric	Description	Equipment	Technique	Authors
Volume	Volume calculation by measuring bladder diameters and applying correction factors	BladderScan BVI94000,	Transabdominal B-mode (2D)	Sheen[5] Vinod[6]
		PadScan Z5,		Zhao[7]
		GE Voluson E8 4–8.5 MHz probe	Transabdominal B-mode (3D)	Nagle[8]
Wall Thickness	Anterior wall average thickness at ≥ 3 sites	3.5 MHz convex probe	Transabdominal B-mode (2D)	De Nunzio[9]
Shape	Calculation of height-width ratios or sagittal perimeters	Philips Purewave CX50	BlaST (Bladder Shape Test) Protocol	Gray[10]
		GE Voluson E8 system 4–8 MHz probe	Transabdominal B-mode (3D)	GlassClark[11] Li[12]
Vibrometry	Lambda wave pulses to measure wall flexibility, movement	Curvilinear transducer, 2.5 MHz	Lambda wave pulses (200–600 µs)	Nenadic[13] Rosen[14]
Elastography	Shear wave correlation of wall thickness to detrusor overactivity	C1-6 XDclear™ probe 1–6 mHz	Shear wave elastography (Logiq™ E9)	Sturm[15]
Compliance	Lambda wave speed correlation with detrusor pressure	Verasonics V1 2.5 MHz probe	Transabdominal B-mode (2D)	Zhang[16]
Biomechanics	Change in perimeter over change in volume	GE Voluson E8 4–8 MHz probe	Transabdominal B-mode (3D)	Nagle[17]
Micromotion	Change in wall thickness over time	Philips Epiq-7 1–5 MHz probe	Transabdominal M-Mode	Nagle[18]

### Bladder Volume

One of the key benefits of UDS, is that the urodynamicist can precisely control the rate of bladder infusion. Thus, the volume at which a bladder “event,” such as an involuntary contraction occurs, can be clearly identified. The problem with attempts at developing non-invasive (i.e. catheter-free) ultrasound UDS technologies is the lack of ability to control for filling volume. The simplest technique to measure bladder volume non-invasively involves a linear estimate of fill rate.[24] For example, if a patient presents with an empty bladder, confirmed with a bladder scanning device, and voids 360 ml after a 2-h study, we can calculate the average fill rate as 180 ml/hour or 3.0 ml/min. If we assume that the fill rate remains constant, we can estimate volume at any time point during the study. Unfortunately, unless a patient is in a state of maximum diuresis, the fill rate is often quite variable.[24].

Researchers have addressed this issue by creating various natural filling hydration protocols. In one of these studies,[25] participants recorded bladder sensations while being asked to void and drink known amounts of water at prompted intervals (every 10 min) to maintain a constant state of maximum diuresis. Heeringa and colleagues showed that perceived bladder fullness and urgency are significantly related to bladder volume.[26] In a more recent study using ultrasound, researchers employed an oral hydration protocol with healthy participants to create a bladder filling method with consistent and reliable results.[5] Other groups have used hydration protocols, and the National Institutes of Health funded Lower Urinary Tract Dysfunction Research Network (**LURN**) study published a detailed protocol describing their technique.[27].

In a study by Sheen and colleagues,[5] the investigators acquired volume measurements using a bladder scanning device every five minutes during filling after a period of oral hydration in normal, healthy volunteers. Bladder scanning measurements were used to calculate real-time bladder volumes during two complete fill-void cycles. Volumes were calculated by fitting a second-order polynomial function to the volume data at each 5% capacity increment of the first (slower) fill and using a linear fill estimate for the second (faster) fill.[5] The investigators found that the initial fill after the onset of oral hydration was a ramp-up phase where the fill rate was accelerating, and the average rate was approximately 7 ml/min. However, maximum diuresis of approximately 15 ml/min was achieved and maintained during the second fill which was found to be reproducible over multiple weekly visits. Importantly, this study details an oral hydration natural filling protocol that could be used as a non-invasive method to estimate bladder volumes either with or without the use of bladder scanning or other bladder ultrasound measurements.

To determine which form of ultrasound provides the most accurate estimate of real-time bladder volume, Vinod and colleagues[6] conducted a study comparing two different forms of ultrasound during oral hydration studies: A bladder scanning device and 3D ultrasound. In this study, end-fill ultrasound volume measurements were compared to voided volumes. Both bladder scanner and 3D ultrasound techniques were found to consistently underestimate voided volumes.[6] However, the data were used to create correction factors based on bladder volume for each modality, as many urology and urogynecology practices utilize different types of ultrasound equipment. Similarly, Zhao and colleagues developed bladder volume correction factors based on bladder shape.[7] The combination of both an oral hydration protocol and defined metrics for accurate estimation of real-time bladder volumes during filling opens the door for future non-invasive urodynamic techniques using ultrasound.

## Bladder Wall Thickness and Bladder Weight

### Assessment of Bladder Outlet Obstruction

The use of ultrasound imaging to quantify bladder wall thickness (**BWT**) and bladder weight (**BW**) has been studied as a screening tool to assess various pathologies including the presence of bladder outlet obstruction and detrusor overactivity. Bladder weight is determined by subtracting luminal from total bladder volume. The technique uses ultrasound to measure the luminal and total bladder radius and assumes a spherical bladder shape.[28] BWT is calculated by taking an average of ultrasound-measured anterior bladder wall thickness over at least three different sites.[29, 30] Investigators found that BWT measurements are highly variable during early filling and remain stable after 50% bladder capacity is achieved.[31] A recent metanalysis by Chen and colleagues[32] examined the results of ultrasound BWT and BW from more than 1800 patients in 16 separate studies which compared these ultrasound metrics to the diagnosis of bladder outlet obstruction. They reported an overall BWT sensitivity of 68% and specificity of 91%. For BW, the sensitivity was 88% and the specificity was 81%, suggesting that both BWT and BW may have potential efficacy for the non-invasive diagnosis of bladder outlet obstruction in men exhibiting lower urinary tract symptoms.

Furthermore, the ultrasound measurements of BWT correlate positively with symptoms scores and clinical parameters in patients with benign prostatic hyperplasia.[33] Lastly, BWT has been found to accurately predict responses to medical/surgical treatment of lower urinary tract symptoms resulting from benign prostatic obstruction.[33] However, the lack of standardization and participant diversity among the available studies requires additional larger, prospective studies prior to widespread implementation into clinical practice.

Overall, the ultrasound evaluation of BW and BWT appears to be simple, highly accurate and non-invasive technique to predict bladder outlet obstruction and to evaluate the clinical outcomes after medical/surgical treatments for benign prostatic hyperplasia. The implementation of these techniques and their standardization will hopefully lead to improved diagnostic algorithms for men with lower urinary tract symptoms and could potentially reduce the requirement for pressure flow-studies.

### Assessment of Detrusor Overactivity

Another application of BWT is the non-invasive identification of detrusor overactivity. In one study,[29] investigators assessed whether BWT measured through ultrasound could replace other screening tools as a noninvasive test for detrusor overactivity. In the study, women underwent both invasive transvaginal ultrasound and urodynamic studies to determine which had a higher detection rate. Transvaginal ultrasound showed a low sensitivity (43%) in women with OAB, indicating limited utility of this technique in this context.[29].

### Assessment of Detrusor Underactivity

Studies have shown that ultrasound might be feasible in the diagnosis of detrusor underactivity. One study involving 33 patients compared BWT with possible filling volume[34] and showed an 87% accuracy in detecting detrusor underactivity. Indeed, all men in the study with a bladder capacity greater than 445 mL and BWT less than 1.23 mm were found to have detrusor underactivity.[34] Building on this work, another group used suprapubic ultrasound to measure BWT and created a nomogram to improve future clinical accuracy.[9] Ultrasound in conjunction with linear regression identified a significant difference in BWT between those with detrusor underactivity and control patients demonstrating a possible clinical benefit when assessing individuals affected by this disorder.[9].

## Bladder Shape

### Why shape matters

**Fig. 1:**
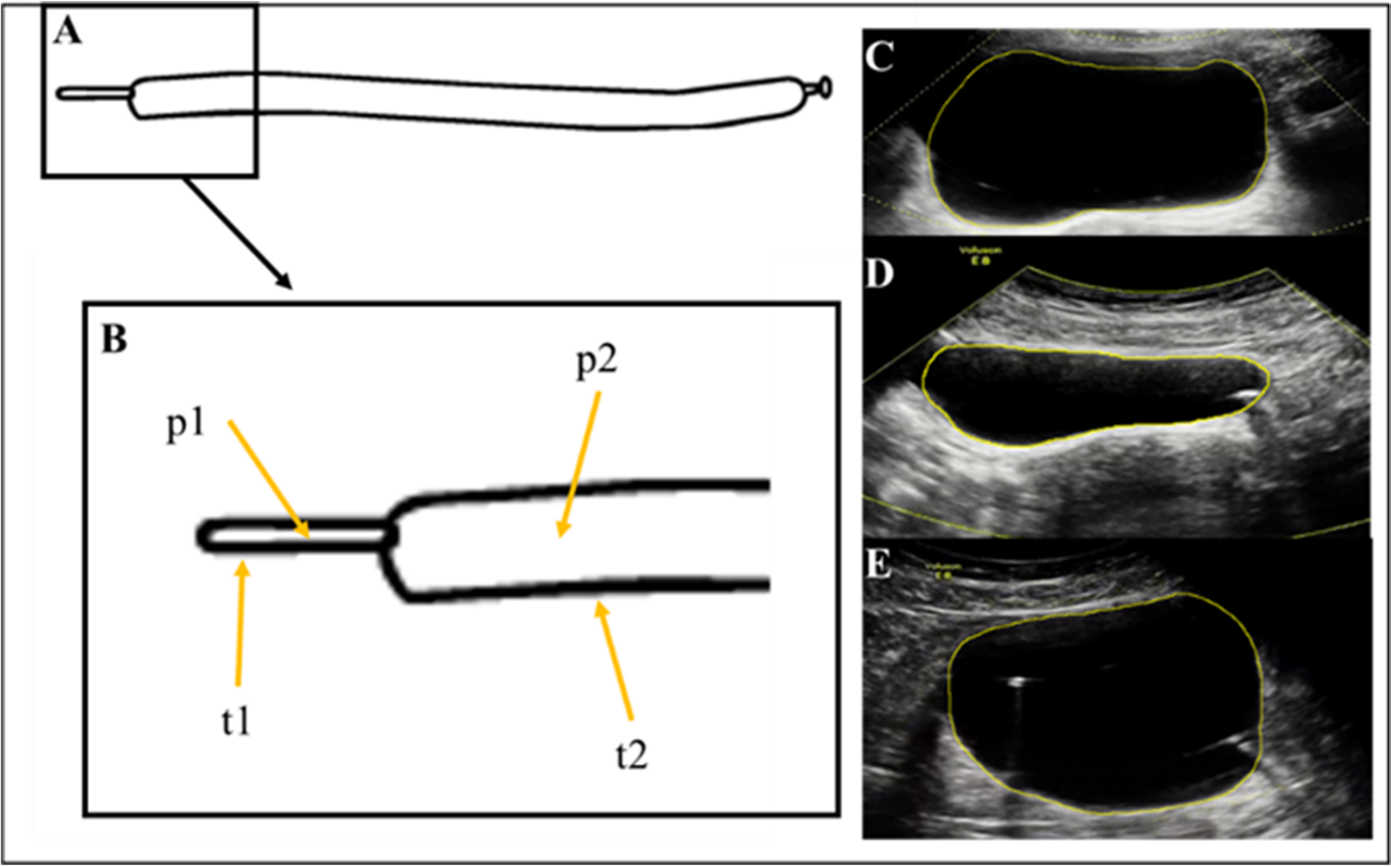
In a partially inflated balloon (**A**, **B**), pressure (p) is constant throughout (p1 = p2). However, due to differences in shape, wall tension varies (t1 < t2). This balloon analogy demonstrates how wall tension can vary based on bladder location. Because many bladders have irregular shapes as shown in **C**, **D**, **E**. Therefore, bladder shape matters, but 3D shape can't be assessed using UDS (but can be assessed with ultrasound imaging)

According to the Law of Laplace, the wall tension (**T**) in the bladder is related to the radius (**R**) of the organ multiplied by the internal fluid pressure (**P**): T ∝ PR. Therefore, any deviation from a bladder's unconstrained shape would create regions of increased wall tension during filling and could lead to heightened sensation. Picture a balloon (Fig. 1). When the balloon is inflated, it has a wide body with high wall tension and a narrow tail with low wall tension. Therefore, in biomechanical terms, shape matters.[35, 36] This is somewhat intuitive; for example, a woman in late-stage pregnancy or with large fibroids may have a pancake-shaped or distorted bladder due to compression. A child with severe constipation will often present with urinary frequency and urgency. A patient with prior pelvic surgery and adhesive bowel disease may have constrained bladder filling, forcing the bladder to assume an irregular shape. Again, shape matters.

Furthermore, tension-sensitive afferent nerves in the bladder[37] likely contribute to the sensation of bladder filling which can subsequently lead to urgency. Acute or chronic changes in a bladder’s material properties (elasticity) or geometry (volume and shape) may also affect bladder wall tension, and therefore, the activation of nerves responsible for bladder sensation. Several imaging techniques, including magnetic resonance imaging and computed tomography, have been used to examine bladder shape.[38] Normal bladders vary in shape,[11, 12, 39] and bladder compliance is related to shape.[35, 36] Because a clinical urodynamic study measures pressures, but not shape, variations in bladder shape and the corresponding localized variations in wall tension and tension sensor output cannot currently be evaluated. Nagle et al.[17] demonstrated how ultrasound urodynamics can be used to measure biomechanical parameters during filling, including strain, tension, stress, and elastic modulus of the bladder wall.

**Fig. 2:**
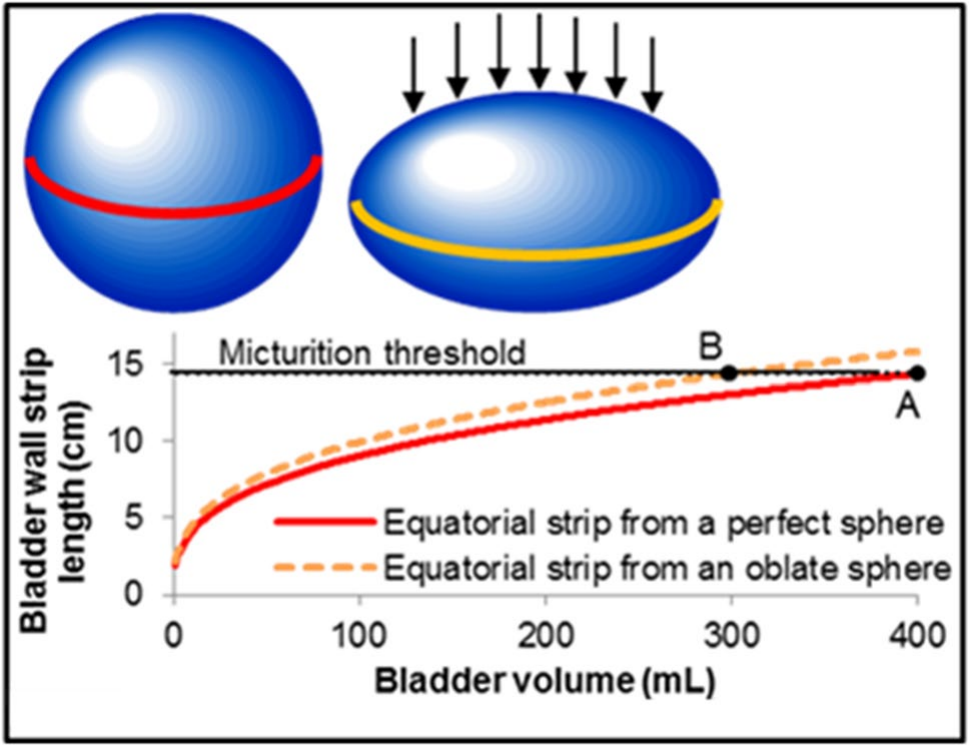
In this illustration, a spherical bladder reaches its micturition threshold at 400 mL with an equatorial wall strip length equal to 1/2 the circumference, or 14.4 cm (red strip, point **A**). If the bladder is contrained by 25% in one radial direction to form an oblate sphere (arrows), it reaches the same threshold strip length (14.4 cm) and therfore approximately the same tension sensor load at a dramatically lower volume of $\backsim$ 300 mL (orange strip, point **B**)

As shown in Fig. 2, bladder filling that is constrained by external forces, as seen acutely in the setting of positional changes during cystometrics,[40] and chronically, in the setting of pregnancy,[41] obesity,[42] or constipation,[43] can increase the load on tension-sensing nerves and lead to a reduced micturition threshold volume.

### How to measure bladder shape

Accepting that bladder shape may be an important driver of bladder sensation and contractility and may relate to disorders of urinary urgency, a clear research and clinical objective for urodynamics is the development of tools that can accurately determine aspects of bladder shape. Ultrasound can be used for this purpose. In a comparative-fill urodynamic study, ultrasound was used to determine changes in volume for participants with OAB.[8] During analysis, there were three different image calculations used: 3D volume, 2D spheroid geometry, and 2D spheroid geometry with a correction factor.[44] 3D bladder ultrasound was found to be the most precise.[8] During continuous filling, 3D ultrasound was more likely to recognize abnormally shaped bladders than either of the 2D counterparts.[8] This showed that 3D bladder ultrasound would offer the best imaging modality moving forward for detecting and measuring irregularities and changes in bladder shape.

### Bladder Shape as a Diagnostic Tool

Although the bladder has often been modeled as a sphere or spheroid,[35, 36, 45] imaging has revealed that bladder shape is highly variable and asymmetrical. Studies have demonstrated banana-like shapes in people with pelvic lipomatosis[46] and that bladder shape changes during filling which, as a consequence, can alter its function.[47] Multiple studies have evaluated bladder shape as a potential diagnostic tool for OAB. In a study performed during urodynamics and natural filling, the investigators used ultrasound to determine bladder shape differences between asymptomatic patients and OAB patients.[10] They identified changes in bladder shape associated with detrusor muscle contraction, showing an increase incidence of ellipsoid shapes in participants with OAB. This was found mainly in the transverse plane. In contrast, a prospective study using 3D ultrasound showed that bladder shape irregularities were most consistently identified in the sagittal plane, and that these irregularities were also associated with OAB.[12].

In another study of women with and without OAB, transabdominal 3D ultrasound was used to determine height-to-width ratios of the bladder in multiple planes as a measure of shape.[11] Ratios were compared at low (20% capacity) and high volumes (100% capacity) to calculate strain. In both groups, change in height during filling was more pronounced than changes in other planes, indicating that bladder filling is not spherical but mainly occurs in the vertical (craniocaudal) plane. However, the key difference was that OAB patients were more likely to have non-spherical bladder shapes than controls. This study suggested that irregular bladder shapes may be a causative mechanism of OAB and that a shape-mediated OAB phenotype could be identified, non-invasively, using ultrasound during bladder filling. Future clinical tests using ultrasound will require an automated method for shape and volume measurement that is both reliable and reproducible, given that most aspects of bladder shape change as the bladder fills.

## Bladder Vibrometry and Elastography

### Vibrometry

As an alternative approach to urodynamics, investigators have used ultrasound with Lamb wave pulsation to determine the flexibility and movement of bladder wall tissues.[13, 48] With this technique, the ultrasound device emits a wavelike pulsation to move bladder wall tissue, and this movement is used to determine tissue properties including elasticity.[13] To calculate elasticity, assumptions were required to estimate bladder wall thickness and bladder curvature. In subsequent studies[14] bladder vibrometry was used as tool to identify detrusor overactivity in individuals with neurogenic bladder dysfunction. This study created a detrusor overactivity index which correlated vibrometry measurements with urodynamically identified detrusor overactivity, demonstrating the potentially feasibility of this technique as a non-invasive alternative to UDS.

### Elastography

Elastography uses ultrasound shear waves to measure tissue stiffness based on the response to applied stress. This novel technique has been used to measure increased bladder wall fibrosis (increased thickness) associated with detrusor overactivity.[15] The authors of this study propose that the bladder becomes less compliant due to increased detrusor activity. As a result, increased shear wave stress is needed to cause movement in the anterior bladder wall.[15] The investigators showed a strong positive correlation between both measured anterior and posterior bladder wall shear wave stress and pressure, showing proof of principle that ultrasound elastography could potentially be used as a non-invasive measure of detrusor overactivity. Another more recent study used ultrasound elastography of the bladder in the assessment of patients with known neurogenic bladder dysfunction.[49] Using urodynamic metrics of detrusor overactivity and poor compliance as markers of neurogenic bladder dysfunction, the authors found that shear wave velocity was increased in approximately 80% of patients with neurogenic bladder. They conclude that bladder elastography has high specificity for identification of UDS parameters associated with neurogenic bladder dysfunction.

## Bladder Compliance and Biomechanics

### Compliance

A recent study completed by Zhang and colleagues[16] used ultrasound bladder vibrometry to measure bladder wall compliance. In this urodynamic study, Lambda wave speed correlated with both intravascular and detrusor pressure. Furthermore, a compliance index, created using Lambda wave speed and filling volume, was comparable to standardized UDS compliance measurements (change in volume over change in pressure). Bladders were considered non-compliant with a vibrometry index of less than 100 mL·s^2^·m^−2^. The resulting sensitivity (86.4%) and specificity (71.9%) demonstrated acceptable accuracy as an alternative, and non-invasive, measurement of detrusor compliance.

### Bladder Biomechanics

In a study conducted by Nagle and colleagues,[17] the investigators performed ultrasound in conjunction with UDS to determine bladder biomechanical parameters including pressure, volume, strain, tension, and stress. While many of these measurements required direct measurements of intravesical pressure, perimeter strain (change in perimeter over change in volume) only required ultrasound imaging and represents a possible assessment tool for OAB. In conjunction with UDS, the authors propose that ultrasound could be used as a future tool to identify different subtypes of OAB.

### Bladder Compression and Provoked Compliance

Dynamic elasticity describes a material property of the bladder wall whereby wall tension, and therefore sensation, can be acutely lowered by passive filling/emptying and restored by contractile activity.[50, 51] In fact, dynamic elasticity has been identified using comparative-fill UDS.[51, 52] The challenge is that comparative-fill UDS are difficult, invasive and time-consuming. To address this issue, bladder compression has been studied in an isolated porcine bladder model and has been shown to acutely alter dynamic elasticity.[53, 54] Consequently, abdominal compression with an ultrasound probe provides a novel method to acutely alter bladder shape (increase strain) and an imaging modality that can be used to quantify the degree of strain. When combined with UDS, changes in bladder shape due to compression divided by changes in pressure have been used to define a new provoked compliance metric.[55] Ultimately, provoked compliance may represent a useful alternative to standard measures of bladder compliance.

## Bladder Wall Micromotion

In a study by Nagle and colleagues, M-mode ultrasound of the anterior bladder wall was used to identify both the outer and luminal edges prior to micromotion calculation.(18) Micromotion was defined as greater than 0.1 mm change in wall thickness after controlling for macro-movements such as breathing. The results demonstrated that approximately 40% of OAB women were found to have ultrasound-identified micromotion, but no asymptomatic women were found to have micromotion. In addition, micromotion correlated strongly with the presence of involuntary detrusor contractions, indicating that this technique has potential utility as a non-invasive detector of micromotion.

## Conclusion

Ultrasound is a promising tool that has the potential to supplement or even replace invasive UDS in the future. Multiple ultrasound UDS research studies have been conducted. Nevertheless, the results have mainly been limited in scale and, as yet, there has not been widespread clinical adoption of any new techniques. Research is ongoing regarding which bladder parameters will ultimately prove useful as reliable, reproducible and discriminatory markers for functional bladder pathology. Currently, bladder wall thickness, shape, vibrometry, elastography, compliance, biomechanics, and micromotion are being investigated as novel ultrasound UDS techniques. Continued research with larger, comparative multi-institutional studies will be required before ultrasound UDS becomes accepted as a diagnostic tool in the assessment, management and understanding of functional bladder disorders.

## Data Availability

No datasets were generated or analysed during the current study.
